# Strategies and Bottlenecks in Hexaploid Wheat to Mobilize Soil Iron to Grains

**DOI:** 10.3389/fpls.2022.863849

**Published:** 2022-04-29

**Authors:** Anil Kumar, Gazaldeep Kaur, Palvinder Singh, Varsha Meena, Shivani Sharma, Manish Tiwari, Petra Bauer, Ajay Kumar Pandey

**Affiliations:** ^1^Department of Biotechnology, National Agri-Food Biotechnology Institute, Mohali, India; ^2^CSIR-National Botanical Research Institute, Lucknow, India; ^3^Institute of Botany, Heinrich Heine University Düsseldorf, Düsseldorf, Germany; ^4^Cluster of Excellence on Plant Sciences, Heinrich Heine University Düsseldorf, Düsseldorf, Germany

**Keywords:** iron, wheat, transporter, grains, phloem, basic helix loop helix

## Abstract

Our knowledge of iron (Fe) uptake and mobilization in plants is mainly based on *Arabidopsis* and rice. Although multiple players of Fe homeostasis have been elucidated, there is a significant gap in our understanding of crop species, such as wheat. It is, therefore, imperative not only to understand the different hurdles for Fe enrichment in tissues but also to address specifically the knowns/unknowns involved in the plausible mechanism of Fe sensing, signaling, transport, and subsequent storage in plants. In the present review, a unique perspective has been described in light of recent knowledge generated in wheat, an economically important crop. The strategies to boost efficient Fe uptake, transcriptional regulation, and long-distance mobilization in grains have been discussed, emphasizing recent biotechnological routes to load Fe in grains. This article also highlights the new elements of physiological and molecular genetics that underpin the mechanistic insight for the identified Fe-related genes and discusses the bottlenecks in unloading the Fe in grains. The information presented here will provide much-needed resources and directions to overcome challenges and design efficient strategies to enhance the Fe density in wheat grains.

## Introduction

Iron (Fe) is an essential micronutrient for all life forms that participate as catalytic cofactors in several vital processes; including phosphorylation, photosynthesis, and chlorophyll biosynthesis in plants (Yadavalli et al., 2012; Kroh and Pilon, 2020; Schmidt et al., 2020). The connection between Fe deficiency and its effect on human health is an important research subject nowadays. Improving the grain Fe content has been considered a unique way to tackle the hidden hunger and Fe deficiency ailments in humans (micronutrient biofortification). Wheat is an important source of energy nutrition in developing countries and has been a target crop for addressing multiple nutritional traits, including micronutrient enhancement, such as Fe and Zinc (Zn) (Borg et al., 2012; Sui et al., 2012; Chattha et al., 2017; Sazawal et al., 2018). The limited genetic variation for Fe content and its low bioavailability in improved adapted wheat varieties have posed challenges to the breeders (Velu et al., 2014). In addition to this, multiple bottlenecks have been identified for the uptake and mobilization of Fe in plants. After the uptake of Fe, it has to be distributed into plant organelles through various transporters, tissues *via* the cell-to-cell communication network and different organs through phloem and xylem mediated long-distance transport systems. Research on Fe homeostasis has been largely focused on the model plant *Arabidopsis thaliana*, which represents a non-graminaceous plant system. Earlier, the lack of wheat gene resources and information about detailed mechanisms involved in Fe homeostasis was a major obstacle for devising biofortification approaches. Therefore, it is imperative to discuss and explore new target areas that could be conserved or specific to wheat crops to deploy biotechnological routes to generate high Fe in grain.

Herein, we primarily provide a glimpse of the genetic components involved in the uptake and mobilization of Fe from roots to the grains and the potential gene targets. Although Fe loading in cereals’ grain is a multistep process, few reliable strategies for grain micronutrient enrichment can be devised by drawing parallels with other plants, including the model plant *Arabidopsis* and *Oryza sativa*. Some of these genetic components have been specifically identified and reported recently in wheat (Kaur et al., 2019; Wang M. et al., 2019). In the present review, some of the important candidate genes that could be used by biotechnological interventions are described and innovative measures have also been discussed. We have provided an insight into the comparative account of genes involved in Fe homeostasis from well-studied plants, such as *Arabidopsis* and *O. sativa*, and presented an overview of the gene regulatory network. We have also emphasized the anatomical bottlenecks and the key areas that need immediate attention to finally generate new genotypes with high Fe in grains for developing viable and sustainable approaches to overcome low Fe in wheat grain. In turn, the challenges of grain Fe enrichment and bottleneck through genetic engineering are discussed in this review with emphasis on possible solutions as well.

## Conserved Molecular Components of Fe Uptake in Wheat

Fe is found to be the fourth most abundant metal in soil. However, the majority of Fe in the soil is present in the form that plants cannot assimilate. In aerobic soil, Fe concentration and bioavailability depends on inorganic forms, such as oxides, hydroxides, and phosphate. Trivalent form (Fe^3+^) is the predominant form of Fe present in soil and the alkaline pH of soil favors the formation of hydrous Fe^3+^ oxides (a solid and insoluble state of Fe), hence becomes inaccessible to the plants (Lindsay, 1991). To acquire this form, strategy I plants reduce Fe^3+^ to ferrous (Fe^2+^) and then facilitate its transport whereas strategy II plants export phytosiderophores (PS) that chelate Fe^3+^ and make this form bio-available to plants (Kobayashi and Nishizawa, 2012).

Wheat utilizes a chelation-based strategy (strategy-II) of Fe uptake. The Fe transport mechanism in wheat is coupled with the production of Fe chelators, such as 2-deoxymugineic acid (DMA) and mugineic acid (MA), and their subsequent secretion into the rhizosphere. Among these PS, DMA is the predominating Fe chelator secreted by wheat roots (Tolay et al., 2001) into the rhizosphere through the transporter of mugineic acid (*TOM*) and facilitates the formation of PS-Fe^3+^ complex (Nozoye et al., 2011). This bioavailable PS-Fe complex is further taken up in the plant root through yellow stripe-like transporters (*YSL*; Curie et al., 2001). The gene families that constitute the strategy-II pathway of Fe transport have been characterized in wheat. The wheat genome encodes for 21 *NAS*, 6 *NAAT*, 3 *DMAS*, 5 *TOM*, and 26 *YSL* genes (Bonneau et al., 2016; Beasley et al., 2017; Kumar et al., 2019; Sharma et al., 2019). Table 1 summarizes the number of genes reported in *Arabidopsis*, rice, and wheat. The expression analysis of these identified genes under Fe limiting conditions showed significant induction in wheat root and shoot (Kaur et al., 2019; Wang M. et al., 2019). Although the differential expression pattern of these genes indicates their potential involvement in Fe transport, their site of action and functional studies in wheat are yet to be explored. Interestingly, the number of genes for each family shows the highest number of genes in hexaploid wheat compared to rice and other crops.

**TABLE 1:** Putative orthologs/homologs and their number of genes for the Fe uptake reported from *Arabidopsis*, rice, and wheat.

Gene	Plant	Number	Potential function	References
NAS	*Arabidopsis*	3	NA biosynthesis	Higuchi et al., 1999
	Rice	6		Inoue et al., 2003
	Wheat	21		Bonneau et al., 2016
NAAT	*Arabidopsis*	–	Intermediary enzyme for NA biosynthesis	–
	Rice	6		Cheng et al., 2007
	Wheat	6		Beasley et al., 2017
DMAS	*Arabidopsis*	–	Intermediary enzyme for NA biosynthesis	–
	Rice	1/(1*)		Nozoye et al., 2004; Bashir et al., 2006
	Wheat	3		Beasley et al., 2017
TOM	*Arabidopsis*	–	MAs efflux transporters	–
	Rice	3		Nozoye et al., 2011, 2015
	Wheat	5		Sharma et al., 2019
YS/YSL	*Arabidopsis*	8	Uptake/Long Distance transport/remobilization of metal-chelates	Curie et al., 2001
	Rice	18		Gross et al., 2003
	Wheat	26		Kumar et al., 2019

(1*) Putative OsDMAS2.

Interestingly, transcriptome data showed the presence of strategy I components, including Iron-Regulated Transporter 1 (*IRT1*) and Ferric Reduction Oxidase (*FRO*) genes, in wheat. The functional studies of *IRT1* and *FRO2* in *Arabidopsis* and other crops are already known for Fe transport *via* strategy I (Eide et al., 1996; Wu et al., 2005). However, rice, barley, and other prominent graminaceous cereals retain both strategies for Fe acquisition simultaneously (Ishimaru et al., 2006). The expression analysis showed significant induction of *IRT1* orthologs in wheat under Fe limiting conditions, whereas strategy 1 genes were unaltered (Kaur et al., 2019). Our previous study also showed that as many as 51 genes encoding putative *IRT/ZIP* Fe and Zn transporters are present in wheat (Kaur et al., 2019). This may suggest that the *IRT1* genes could function independently of strategy II component, which is the predominant route of Fe acquisition in wheat.

Moreover, the production and release of various metabolites are paired with changes in root phenotype under Fe deficiency in wheat. In the recent past, numerous reports have described that the exudation of phenylpropanoids and flavin-derived metabolites from roots are essential for efficient Fe uptake in calcareous soil and alkaline conditions (Rodríguez-Celma et al., 2013; Schmid et al., 2014; Sisó-Terraza et al., 2016). These derivatives may help in the solubilization of (Fe^3+^) Fe precipitates and subsequent reduction of Fe^3+^ to Fe^2+^ for direct import (Mladìnka et al., 2010). Metabolic analysis of wheat under Fe limiting conditions revealed the enhanced production of organic acids and polyhydroxy acids, such as fumarate, malonate, succinate, and xylofuranose, along with PS release in wheat (Garnica et al., 2018; Kaur et al., 2019).

## Interaction of Fe With Phosphorus and Sulfur

Despite the varying concentrations of nutrients in soil, plants maintain a cellular nutrient balance for optimal growth as well as avoid toxic effects. This is achieved by the interwoven nutrient homeostasis and synergistic/antagonistic interactions among the nutrients. Any change in soil nutrients is efficiently sensed by the root, which initiates a transcriptional network that eventually helps the plant to deal with the deficiency/excess response. Depending on the charge, some nutrients interact with others in the rhizosphere and determine their bioavailabilities. For instance, phosphorus (P) and sulfur (S) display interconnected homeostasis with Fe in the rhizosphere (Hirsch et al., 2006; Ward et al., 2008). P exists in many chemical forms in soil, but plants acquire mainly inorganic phosphate (Pi) and organic phosphorus (Po) for their growth and development. The strong interaction between Fe and Pi in soil forms precipitates, affecting the availability of both nutrients to the plant. It has been observed that a high-affinity root Fe^2+^ uptake system which is usually activated under Fe-deficiency, is also induced under excess Pi environment (Ward et al., 2008). Similarly, under Pi-deficient conditions, Arabidopsis accumulated more Fe and heavy metals (Misson et al., 2005; Hirsch et al., 2006). Studies showed that increased accumulation of Fe in Pi-deficient medium causes Fe-toxicity leading to primary root inhibition (Ward et al., 2008; Ticconi et al., 2009). In Arabidopsis, the increase in Fe availability after Pi-deficiency was thought to be correlated at the molecular level by induction of AtFER1, which encodes ferritin (a Fe storage protein) (Hirsch et al., 2006). However, other ferritin genes, such as AtFER3 and AtFER4 lack PHR1-binding sites, in their promoters and are induced under high Fe but non-responsive to Pi-starvation (Petit et al., 2001; Bournier et al., 2013). In hexaploid wheat, the physiological effects were accounted for the molecular changes that involve the regulation of a subset of downstream signaling genes potentially cross-regulated by Fe and Pi (Kaur et al., 2021). The data set from this study reveals that PS release and metabolic pathway for phenyl-propanoid plays an important role for Fe mobilization in the roots under single and dual deficiency of these nutrients (Kaur et al., 2021).

By contrast, many studies have reported a synergistic interaction between Fe and S in the rhizosphere. The S and Fe are highly essential for the optimal functioning of chlorophyll biosynthesis, thereby controlling the plant’s photosynthetic ability through modulating the assembly of Fe-S cluster proteins (Couturier et al., 2013). The genes involved in S assimilation, namely TdSultr1.1, TdSultr1.3, TdAPR, and TdSiR showed similar overlapping expressions under Fe and S deficiency in the durum wheat (Ciaffi et al., 2013). Fe uptake and assimilation are hampered under the S depleted condition (Astolfi et al., 2003, 2018; Ciaffi et al., 2013). Further, when plants are subjected to an S limiting environment, a reduction of NA in the shoot is observed even under Fe sufficient conditions resulting in the low mobilization of micronutrients (Fe and Zn) toward the sink tissue. Thus, it is conclusive that elemental interactions in the rhizosphere also create multiple nutrient deficiencies for plants and dramatically affect plant growth, and could modulate the Fe availability in roots for its directional uptake.

## Transcriptional Regulation of Fe Homeostasis in Wheat

**FIGURE 1:**
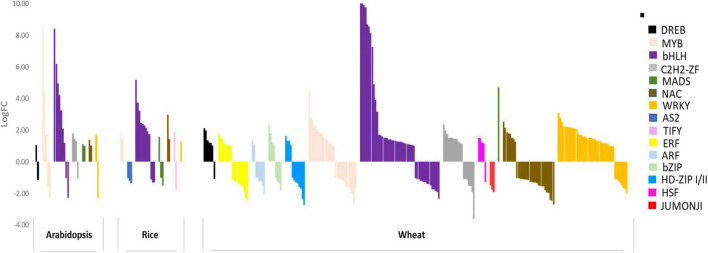
TF subfamilies differentially expressed (DE) in response to Fe starvation in *Arabidopsis*, rice, and wheat. Expression bar plot representing transcription factor subfamilies differentially expressed in response to Fe starvation in *Arabidopsis*, rice and wheat. *X*-axis depicts the TF genes in respective species and *Y*-axis shows the log fold change (logFC) for the respective genes under Fe starvation with reference to the respective control samples. TF subfamilies are color-coded according to the legend on the right. Respective datasets were downloaded and analyzed for DEGs using the Kallisto-DESeq2 pipeline after getting high-quality, adapter trimmed reads using Trimmomatic. The DEGs for respective species were annotated for TF subfamilies using Mercator4 v2.0 (Schwacke et al., 2019) (TF subfamilies with only 1 gene DE in At and rice; less than 5 genes DE for wheat are not depicted in this graph).

Although few molecular components, including transporters, are largely known, the entire transcriptional regulation of genes involved in Fe deficiency response remains elusive mainly in wheat. Under Fe-deficiency, hexaploid wheat revealed a perturbation of the sub-set of transcription factors (TFs), including *basic helix-loop-helix* (*bHLH*), *basic leucine zipper* (*bZIP*), *WRKY*, *C2H2*, *MYB*, *NAC*, and *homeobox-leucine zipper* (Figure 1). Among these, *bHLHs* were found to be the most predominant TFs suggesting their conserved role in regulating Fe deficiency-related responses (Kaur et al., 2019). The role of *bHLHs* mediated transcriptional regulation has been extensively investigated in *Arabidopsis* and to some extent in rice. Given the large role of bHLH in Fe-homeostasis, we will be focusing on the homologs of the candidate bHLH genes in wheat. We have summarized the paralogs of bHLHs in wheat-related with Fe-homeostasis based on orthologous sequence similarity with *Arabidopsis* and rice (Table 2). The bHLH family in plants is clustered into 26 different groups out of which six sub-groups are reported to regulate Fe homeostasis or deficiency response in *Arabidopsis* (Pires and Dolan, 2010). The FIT (Fer-Like Fe Deficiency-Induced Transcription Factor), a homolog of the FER transcription factor, has been demonstrated to play a central role in adapting and controlling the amount of Fe uptake in *Arabidopsis* (Ling et al., 2002; Colangelo and Guerinot, 2004; Jakoby et al., 2004; Yuan et al., 2005; Mai et al., 2015; Schwarz and Bauer, 2020). The FIT is shown to interact with members of the bHLH subgroup Ib (bHLH38, 39, 100, 101) leading to stabilization of FIT and activation of other subgroup Ib bHLHs (Naranjo-Arcos et al., 2017; Cui et al., 2018; Trofimov et al., 2019). These transcriptional networks, in turn, provide the adaptive and sustainable condition for regulating the expression of multiple downstream genes committed for Fe uptake and mobilization in plants (Mai et al., 2016; Schwarz and Bauer, 2020). Further, *OsIRO2*, a Fe related bHLH TF, is thought to be a master regulator of Fe deficiency response in rice and five orthologs of *IRO2* are present in the hexaploid wheat genome (Table 2; Ogo et al., 2007). *IRO2* and *OsbHLH156* regulate the expression of a subset of root-specific Fe deficiency responsive genes, thus helping Fe mobilization. Feedback regulation of FIT can also be tightly controlled by other bHLH TFs as FIT interaction with IVa subgroup members (bHLH18, bHLH19, bHLH20, bHLH25) leads to proteasomal degradation (Cui et al., 2018). The MYC2, a bHLH of subgroup IIIe, regulates the expression of the other genes representing the IVa subgroup (Li et al., 2016; Cui et al., 2018). The bHLH121/URI (member of IVb subgroup) and bHLH034, bHLH104, bHLH105/ILR3, and bHLH115 (members of IVc subgroup) were shown to be involved in the regulation of Fe homeostasis in both rice and *Arabidopsis* (Li et al., 2016; Liang et al., 2017; Wang et al., 2017; Kim et al., 2019; Tissot et al., 2019; Gao et al., 2020). Similarly, *OsbHLH057/PRI4*, *OsbHLH058/PRI2*, *OsbHLH059/PRI3*, and *OsbHLH060/PRI1* act upstream to bHLH genes namely *OsIRO2*, *PYE*, and *IRO3* by binding to their promoters under Fe deficiency (Zhang et al., 2017, 2020; Kobayashi et al., 2019). It is important to mention that bHLHs do not always positively activate Fe homeostasis-related genes expression but *AtPYE* and *OsIRO3* are characterized as negative regulators of certain Fe-deficiency-induced genes (Long et al., 2010; Zheng et al., 2010). *OsIRO3* was found to repress *OsIRO2* transcripts to regulate Fe uptake in rice (Long et al., 2010; Zheng et al., 2010).

**TABLE 2:** Fe homeostasis-related regulatory bHLH protein from *A. thaliana* and their homologs from rice and wheat.

bHLH (subfamily)	bHLH name (*A. thaliana*)	Gene ID (*A. thaliana*)	Homolog in rice	Homolog in wheat
Ib	AtbHLH038 (ORG2)	AT3G56970	OsIRO2	TraesCS2A02G515300 (TabHLH452) TraesCS2D02G517000 (TabHLH453) TraesCS3A02G489600 (TabHLH462) TraesCS3B02G550000 (TabHLH460) TraesCS3D02G495600 (TabHLH461)
	AtbHLH039	AT3G56980		
	AtbHLH100	AT2G41240		
	AtbHLH101	AT5G04150		
IIIa	AtbHLH029 (FIT)	AT2G28160	OsbHLH156	TraesCS2A02G281200 (TabHLH311) TraesCS2B02G298600 (TabHLH312) TraesCS2D02G280100 (TabHLH313)
IIIe	AtbHLH006 (MYC2)	AT1G32640	OsbHLH009	TraesCS1A02G193200 (TabHLH183) TraesCS1B02G208000 TraesCS1D02G196900 (TabHLH184)
IVa	AtbHLH018	AT2G22750	OsbHLH018	TraesCS4B02G056600 (TabHLH284) TraesCS4D02G056900 (TabHLH285) TraesCS4A02G257900 (TabHLH283) TraesCS4A02G408800 (TabHLH260) TraesCS4D02G019100 (TabHLH265) TraesCS4A02G292700 (TabHLH266) TraesCS4D02G018800 (TabHLH268) TraesCS4B02G020700 (TabHLH267)
	AtbHLH019	AT2G22760		
	AtbHLH020 (NAI)	AT2G22770		
	AtbHLH025	AT4G37850		
IVb	AtbHLH047 (PYE)	AT3G47640	OsIRO3	TraesCS2B02G095900 (TabHLH417) TraesCS2D02G079100 (TabHLH418) TraesCS4B02G125400 (TabHLH419)
IVc	AtBHLH034	AT3G23210	OsbHLH057 (OsPRI2) OsbHLH058 (OsPRI2) OsbHLH059 (OsPRI3) OsbHLH060 (OsPRI1)	TraesCS2B02G240600 (TabHLH406) TraesCS2D02G221200 (TabHLH405) TraesCS2A02G215600 (TabHLH404) TraesCS7A02G307700 (TabHLH407) TraesCS7B02G208000 (TabHLH408) TraesCS7D02G304500 (TabHLH409)
	AtbHLH104	AT4G14410		
	AtbHLH105	AT5G54680		
	AtbHLH115	AT1G51070		

**FIGURE 2:**
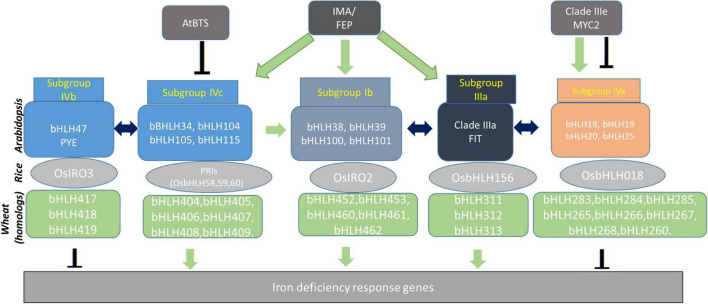
Transcriptional homologs of *Arabidopsis* and rice bHLH proteins reported for their functional activity for Fe homeostasis. The respective homologs from wheat were identified and placed in the network as a probable candidate for function. Their conserved role in Fe-related phenomenon remains to be tested and validated. Many of these bHLH transcription factors are in turn regulated by BRUTUS, IRON MAN: IMA, and bHLH-like MYC2. Schematic arrangement of bHLH dependent interaction and control at the transcription level is indicated by respective arrow signs: Green arrow: transcriptional activation; Inverted T: gene repression; Both side arrow: protein-protein interaction.

Although the role of bHLHs has been systematically characterized in *Arabidopsis* and rice, their distinct or conserved function in wheat is still largely unaddressed. Thus, we decided to generate a transcriptional network of bHLHs in wheat based on their annotation corresponding to homologs in *Arabidopsis* and rice (Figure 2). This network depicts the categorization of the identified bHLHs of wheat into five subgroups and explains how subgroups interact with each other to evoke the gene expression of Fe deficiency (Figure 2). The functional validation of candidate bHLH using genome editing tools or overexpressing wheat lines could be the appropriate way to investigate their role in Fe-homeostasis. It is worthy to mention here that *cis*-regulatory elements (CRE) are the binding sites for TFs regulating the expression of Fe homeostasis-related genes in plants. A machine-learning-based computational method identified novel CREs (∼100 putative CREs) involved in Fe deficiency regulation have been identified in rice and *Arabidopsis* (Schwarz et al., 2020). Similarly, several Fe homeostasis-related orthologs in wheat show the presence of multiple important CREs, including E-box/G-box, in the promoter regions suggesting the occurrence of bHLHs transcriptional hub in wheat (Beasley et al., 2017; Kumar et al., 2019; Sharma et al., 2019, 2020). Altogether, the network of TFs especially the bHLHs hub defines the integration of different regulatory nodes for controlling the downstream target genes involved in Fe uptake and mobilization. Upon functional characterization, these bHLH TFs could be utilized to enhance the mobilization of Fe to foliar parts mainly in grain.

## Biotechnological Routes for Fe Fortification and Accomplishments in Wheat

In the past few years, limited attempts have been made to increase Fe content or its bioavailability in wheat grain using a biotechnological route. The criteria for the selection of genes were mainly based on overlapping functions in rice, maize, barley, and *Arabidopsis*. At this point, it is important to consider the loading of Fe in the grains and enhance its bioavailability for better human nutrition. Therefore, biotechnological routes to enhance these two traits is challenging in crops. Therefore researchers have opted for different strategies to target these traits.

### Targeting Genes for Uptake and Loading in Grains

The first successful report to achieve high Fe in cereals was demonstrated by transferring the soybean ferritin gene driven under rice glutelin-1 promoter resulted in threefold higher Fe content as compared to wildtype rice (cv. Kita-ake) (Goto et al., 1999). Subsequently, many research groups have attempted to improve Fe content in the rice grain using *SoyFer1* in different rice cultivars under the control of different promoters (Drakakaki et al., 2000; Qu et al., 2005; Oliva et al., 2014). The genes involved in Fe uptake like *OsIRT1*, *OsTOM1*, *OsYSLs* (*YSL15*, *YSL2*, and *YSL13*), and long-distance Fe translocation namely *OsNAS1*, *OsNAS2*, *OsNAS3* have been targeted to achieve a significant increase (up to 40 μg/g) of Fe levels in rice (Lee and An, 2009; Lee et al., 2009a,b, 2012; Ishimaru et al., 2010; Nozoye et al., 2011; Díaz-Benito et al., 2018; Zhang et al., 2018). The transfer of genes related to Fe uptake, such as *NAS* and *YSL* from *Hordeum vulgare*, has also been able to increase Fe content in rice varieties (Masuda et al., 2012; Boonyaves et al., 2016; Banakar et al., 2017). A study reported that *OsIRO2*, a gene responsible for Fe utilization and regulatory response, improves growth and yield in calcareous soil as well as accumulated Fe in rice grain (Ogo et al., 2011). Simultaneously, the gene silencing approach was also employed to enhance Fe levels in rice by targeting the intercellular/intracellular transporters comprising *OsVIT1*, *OsVIT2*, *OsYSL9*, and *OsDMAS1* (Zhang et al., 2012; Bashir et al., 2017; Senoura et al., 2017).

Ferritin is a Fe storage protein exclusively targeted to plastids and mitochondria in plants. The ferritin encoding genes are well conserved in the plant kingdom, and the genome of hexaploid wheat contains two ferritin genes. The two separate pioneering studies reported that expression of the ferritin gene in endosperm enhanced Fe up to 1.25–1.35 fold and 1.04–1.64 fold, respectively, in the wheat grain (Borg et al., 2012; Sui et al., 2012). The *Vacuolar Iron Transporter* (*VIT*), which transports Fe into vacuoles, is involved in Fe loading in the *Arabidopsis* seeds (Kim et al., 2006). The overexpression of *TaVIT2* in endosperm caused efficient vacuolar Fe transport in the endosperm which ultimately resulted in a significant ∼2-fold increase in Fe content in wheat grain (Connorton et al., 2017). In an elegant study, Narayanan et al. (2019) combined three genes from the *Arabidopsis* (*VIT*, *IRT1*, and *FER1*) into one expression cassette and developed transgenic lines in cassava (*Manihot esculenta* Crantz), a staple crop in the West African human population. The field data of transgenic lines suggested an elevated Fe and Zn in cassava roots that may benefit the long-awaited goal of improved nutritional status of consumers (Narayanan et al., 2019). Therefore, multiplexing of important genes with different combinations might be a viable option to channelize more Fe in the wheat endosperm. Such a strategy has also been attempted in rice, for instance, overexpression of *AtIRT1*, *PvFerritin*, and *AtNAS1* led to an increment in grain Fe content (2–10 μg/g) (Boonyaves et al., 2016), and a combination of *AtNAS1*, *PvFer*, and *AtNRAMP3* resulted in the rise of Fe from 2 to 13 μg/g (Wu et al., 2019). Using *OsNAS2*, *PvFERRITIN*, and in a combination of both genes, Singh et al. (2017) developed wheat transgenic lines containing enough Fe in grain to meet the recommended dietary allowances for humans (Singh et al., 2017). Moreover, there are several genotypes available with higher Fe and Zn concentrations that could be important resources for breeding (Khokhar et al., 2020). Such wheat lines can also serve as excellent germplasm for the introgression of this trait in elite varieties *via* conventional breeding.

### Enhancing Grain Fe Bioavailability

The cereals accumulate phytic acid (phytate) in the vacuoles which chelate Fe and its metal ions, thereby drastically reducing its bioavailability. The phytate is thus considered an anti-nutrient and low phytate (*lpa*) could be another promising strategy for improving Fe accessibility in cereals (García-Estepa et al., 1999; Bhati et al., 2016). The *lpa* can be attained by repressing or inducing mutation in the genes either responsible for PA biosynthesis or transport to seed. The previous study of our research group identified putative PA-related genes, such as *TaIPK1*, which catalyzes the last step of PA biosynthesis and *TaABCC13* in wheat (Bhati et al., 2014). The silencing of *TaIPK1* using RNAi significantly lowered PA production and led to an increased Fe and Zn content in wheat grain (Bhati et al., 2016; Aggarwal et al., 2018). However, pleiotropic developmental changes, including low crop yield, were observed in wheat RNAi lines. Moreover, Abid et al. (2017) have used *phytase* gene from *Aspergillus japonicus* for the degradation of stored PA in endosperm leading to enhanced bioavailability of both Fe and Zn in wheat (Abid et al., 2017). More recently, using the genome editing-based tool, *TaIPK1* was chosen as a target (Ibrahim et al., 2021). The indel mutations increased the accumulation of both Fe and Zn contents in genome-edited Borlaug 2016 wheat variety wheat compared to the non-edited. Both these studies highlight the IPK1 gene target as a major factor contributing to the bioavailability of Fe in cereal crops.

Furthermore, the constitutive overexpression of the rice *OsNAS2* gene resulted in an increased Fe and Zn content in wheat endosperm. Moreover, the same study also showed an enhanced bioavailability of both metals (Beasley et al., 2019). Recent studies suggest that Fe-bioavailability and its absorption are independent of the enhanced Fe-content in grains. The foremost reason for this is that even though Fe content in the whole seed is increased, it is stored in the aleurone layer which gets removed while milling, leaving only the endosperm for consumption. Therefore, both Fe content and bioavailability should be taken into account while addressing the biofortification of wheat (Eagling et al., 2014; Wright et al., 2021). To further address this, the wheat genome was studied for the identification of the QTLs that could be responsible for both high Fe-content and bioavailability (Wright et al., 2021). Very recently, a two-gene strategy to overexpress VIT-NAS in wheat endosperm showed high Fe concentration and also improves mineral bioaccessibility (Harrington et al., 2022). Such innovative approaches directed at content and its bioavailability is a feasible approach to generate nutrition-rich grains. Our insight about genes and rate-limiting steps of Fe homeostasis in wheat is increasing day by day. The recent characterization of *TaYSL* and *TaZIFL/TaTOM* genes has filled the knowledge gap of the Fe-transport mechanism in wheat (Kumar et al., 2019; Sharma et al., 2019). In addition, transcriptional activators, namely *NAM-B1* and *GPCs* have been found to control Fe and Zn remobilization to sink organs during senescence and grain filling (Uauy et al., 2006; Pearce et al., 2014). We assume that these genes can be potential candidates for gene manipulation/editing efforts either independently or *via* multiplexing to meet the ultimate goal of Fe enriched wheat grain in the future.

## Challenges of Fe Enrichment in Wheat Grain and Integrating Novel Approaches

Being the second most produced cereals in the world, wheat can be the most promising crop for Fe fortification to combat the hidden hunger caused by Fe deficiency in human beings. In the past few years, some efforts have been made in this direction; however, limited success is achieved using genetic engineering. It is noteworthy to mention here that the use of orthologous genes is not sufficient to mobilize Fe in wheat grain up to a certain level. In this section, we describe the main hurdles of increasing Fe content in wheat grain *via* transgenic technology and discuss the future strategies to address limitations as well.

### Loading in the Grains: The Real Bottleneck of Fe Fortification

The main bottleneck of Fe fortification using the transgenic approach appears to be the lack of molecular details of its homeostasis and transcriptional regulators of Fe mobilization in wheat. Upon uptake, micronutrients enter into the root xylem and reach the spikelets where it gets discontinued at the base of the grain (Zee and O’Brien, 1970). At this site, phloem takes a central role to mobilize the macro-and micronutrients by providing passage for unloading into the grain. Studies exhibited that NA is required for phloem unloading and loss of function mutant of *NAS* led to sterility whereas RNAi lines of *NAS* resulted in low nutrients in *Arabidopsis* seeds (Klatte et al., 2009; Schuler et al., 2012).

**FIGURE 3:**
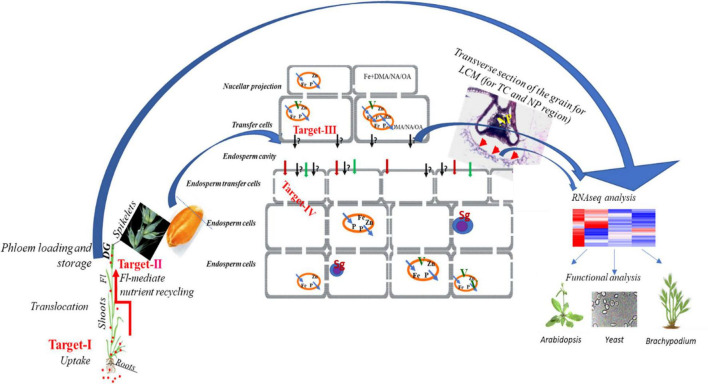
Schematic representation of the primary bottleneck for Fe acquisition, uptake and mobilization in wheat shoot and grain. Multiple target areas have been identified to enhance the mobilization of Fe from soil to roots, from roots to shoots, and subsequently their loading in the grains through specific conducting tissue. The first is referred as Target-I, and Target-II depict the uptake of Fe in roots and then to shoots and eventually loading to the edible grains. In wheat, primarily at this stage, the bottleneck includes the transfer and mobilization of Fe from discontinued xylem to the transfer cells and subsequently to the endodermal cells (Target-III and IV). The target areas Target-III and IV remained unexplored in hexaploid wheat. The area that needs attention is to perform grain or Tissue or single cell-specific-omics approach that will help in identifying key transporters and regulators. These identified key genes could be tested for their functional validation in yeast and subsequent proof-of-function in *Arabidopsis*, *Brachypodium*, or in wheat. The red arrow indicates the process in wheat, and the blue arrow indicates the means to further characterize wheat genes in different model species.

There is an almost complete lack of knowledge about genes, transporters, and transcriptional activators responsible for the root to shoot distribution (xylem loading), phloem loading and unloading of Fe at the time of grain filling in wheat (Figure 3). It should be noted that transfer cells (TCs) play a central role in nutrient distribution by facilitating high rates of transport for apo-/symplastic solute exchange during nutrient loading in phloem and unloading in the endosperm. The TCs found near the endosperm, are specialized cells having unique wall architecture with abundant transporters to enhance membrane transport capacity (Offler et al., 2003). The mechanical removal of TCs reduced *in vitro* photo-assimilate transport rates suggesting that they are responsible for 70–80% of sucrose imported by the filial tissues in wheat (Wang et al., 1995). TCs are also present near fine veins in leaf and nodes where phloem loading takes place and at the interface of maternal and filial tissues which form symplastic solute transport routes in connection with vascular conduits. Even though all imported solutes, including Fe and Zn, have to be transported through TCs in the endosperm cavity, there is a complete lack of understanding about the mode of transport and genes in TCs.

### Exploration of Hitherto Regulators of Fe Homeostasis in Wheat

Recent studies demonstrated that stem nodes in the graminaceous species are important transient sites for the mobilization of micronutrients, including Fe and Zn, to the above-ground organs (Yamaji and Ma, 2014). The genes encoding for plasma membrane-localized efflux transporter for citrate Ferric Reductase Defective like-1 (*OsFRDL1*) and a yellow stripe-like transporter (*OsYSL*) were specifically shown to be expressed in nodes, and demonstrated to be important for the distribution of Fe and Cu in rice grains (Zheng et al., 2012; Yokosho et al., 2016). It has been shown that *Arabidopsis* Ferric Reductase Defective-3 (*AtFRD3*) maintains citrate levels in the xylem of *Arabidopsis*, and mutation in this gene caused an accumulation of Fe in roots (Durrett et al., 2007). However, a distinct anatomical tissue arrangement has been reported in wheat, and multiple modes of efficient distribution of mineral elements have been speculated but the transporters and regulators involved at specific sites are largely unaddressed (Yamaji and Ma, 2014). Studies illustrated that the heterologous expression of *Triticum polonicum* genes namely *TpSnRK2.10* and *TpSnRK2.11* increased Fe accumulation in roots and influenced Fe distribution in *Arabidopsis* (Wang R. et al., 2019). Later studies reported the accumulation of *IRT1* transcripts occurred in SnRK2.2/2.3 dependent manner in *Arabidopsis* (Fan et al., 2014). Furthermore, the plant hormone gibberellic acid (GA) is known to control the expression of *IRT1* and *FRO2 via* DELLA protein in *Arabidopsis* (Matsuoka et al., 2014). The DELLA protein interacts with transcriptional activators, such as *FIT*, *bHLH38*, and *bHLH39*, and limits their transcriptional activities (Wild et al., 2016). The abundance of DELLA is regulated by Fe deficiency which ultimately favors Fe acquisition in roots. It should be worthy to mention here that semi-dwarf alleles of wheat were preferred during the green revolution and modern wheat varieties have been developed showing improved harvest index and resistance to water lodging. In general, modern wheat varieties are less sensitive to GA and contain less Fe in grain as well. Now, it is still unclear whether GA insensitivity and DELLA accumulation may be a reason for the low Fe in grains of commonly growing semi-dwarf modern wheat varieties. Furthermore, Fe interaction with other nutrients is a well-recognized phenomenon in soil for ensuring the bio-availability of Fe to plants which have been described in detail in previous sections of this review. The presence of excess/limited nutrients in the rhizosphere challenges the plant to integrate nutrient signals in the form of genetic shift which finally changes root architecture. The studies have shown that gaseous hormone ethylene is extremely required for the maintenance of primary and lateral root growth as well as Fe homeostasis in *Arabidopsis* (García et al., 2015; Li et al., 2015). Hence, focusing on unconventional hitherto regulators of Fe, such as SnRK, DELLA, components of ethylene signaling and others can boost our endeavor for not only Fe enrichment perspective but also enhanced crop productivity in the Fe deficient soil.

In this line, we describe four research directions that could be emphasized *viz*. Fe uptake, long-distance transport, remobilization, and unloading of Fe in developing wheat grains for dealing with Fe fortification. The schematic representation of these four main target areas has been illustrated in Figure 3. Targets I and II have been proposed to utilize a strategy wherein overexpression of genes encoding for *NAS*, *NAAT*, *DMAS*, *TOM*, and *YSL* could lead to an increase in Fe uptake by enhancing the PS production and release in soil. Next, the collective candidates of Target III and IV cover Fe transport from flag leaf to developing grains. The identification and characterization of transporters and TFs especially comprising Targets III and IV would provide molecular assets to be useful for Fe accumulation through genetic engineering or molecular breeding. Therefore, a concerted effort will be required to identify TCs/tissue-specific genes to cross the bottleneck, and which will serve as potential targets for developing wheat varieties having more Fe in grain.

### Single-Cell RNA Sequencing

The evolution of high-throughput sequencing techniques is going very fast in recent years. The establishment of single-cell RNA sequencing (scRNA-seq) tools revolutionized the molecular studies of developmental processes in any organism. The single-cell transcriptome analysis is very helpful for the spatio-temporal characterization of genes and gene regulatory networks recruited in specific developmental trajectories. Nevertheless, the isolation of single-cell from plant tissues is the most crucial step and is a major constraint for the wide application of scRNA-seq analysis in crops. The technical advancements in this area, such as Drop-seq and InDrop-seq, will gear the implementation of scRNA-seq analysis in plant research. In recent years, scRNA-seq analysis has been successfully implemented to decipher cell type-specific information, for instance, developmental programs leading to meiosis in maize (Nelms and Walbot, 2019) and dynamics of gene expression in the root cells of *Arabidopsis* (Jean-Baptiste et al., 2019). We believe that scRNA-seq analysis of cell files involved in vascular loading and unloading of Fe in sink organs at the time of grain filling stage in wheat can be an ideal way to investigate the gene regulatory network of Fe mobilization. Furthermore, nucellar projection and TCs are the two main cells through which Fe has to pass before loading into the endosperm. The genes that facilitate Fe loading from these cells are still unknown not only in wheat but in model plants as well. Thus, sc RNA-Seq study either under varying Fe conditions or in contrasting Fe accumulating wheat varieties can potentially reveal these missing molecular links that will help in exploring new candidate genes to enhance Fe loading into seed.

### Genome Expression Bias and Fe Homeostasis in Wheat

The modern wheat contains hexaploid genome (2n = 6x = 42) with much complex genome organization. Hence, the investigation of mechanisms, transcriptional regulators, and genes involved in Fe uptake and homeostasis in wheat seems to be a tough task. The wheat genome is derived from three related ancestral genomes from the genera *Aegilops* and *Triticum*. It is of high interest to understand how individual genomes interact with each other for determining the expression of highly related genes in wheat. The phenomenon of genome biasness has been reported earlier in polyploid plants, such as cotton and soybean (Roulin et al., 2013; Yoo et al., 2013). A homeolog-specific differential regulation of genes controlling growth and vigor was also observed in wheat (Akhunova et al., 2010). Subsequent studies pointed the homeolog induction bias under *Fusarium* infection in wheat (Powell et al., 2017). A large expression landscape study was recently conducted suggesting the existence of a tissue-specific co-expression network as well as coordination of homeologs during different developmental stages (Ramírez-González et al., 2018). Such induction bias is not only restricted to growth or biotic stresses but rather plays a significant role during Fe deficiency response in wheat (Kaur et al., 2019). Homeolog induction bias was observed under Fe deficiency for A and B genomes in wheat. Genes contributing toward induction bias comprises metal transporters, Zn transporters, and MYB transcription factors. Furthermore, it has been shown that Fe deficiency response, such as PS release, was more in tetraploid wheat with AABB sub-genome compared to DD genome (Ma et al., 1999). This explains why hexaploid wheat releases high PS simply due to retaining AABB genome biasness. The factors influencing such homeolog induction bias and genome bias remain largely unaddressed. A recent study demonstrates that varying chromatin accessibility of homeologs might be the reason for such genome bias in wheat (Jordan et al., 2020). Therefore, it will be interesting to pinpoint spatio-temporal genome biasness invoked due to Fe deficiency in wheat.

## Conclusion and Future Perspectives

Improving the nutritional quality of cereals is of prime importance and yet challenging. The Fe biofortification in wheat has been an arduous task for many decades with limited success. Therefore, the means to design better strategies for improving Fe uptake by the roots, genetic screening of germplasm showing tolerance under limiting Fe conditions, and combining approaches of classical physiology with the omics data-driven system biological studies will certainly help in undermining the limiting steps involved in Fe remobilization into wheat grain. Also, homeolog specific expression analysis of Fe-regulated genes has revealed the expression biasness for A, B, and D subgenomes (Kaur et al., 2019). These studies have streamlined the selection of candidate gene/s and/or homeologs and also made it much more efficient and rational. The genetic engineering tools, genomic sequence information, gene mining, and functional genomics would help to carry forward the research for developing novel strategies of micronutrient enhancement in wheat. Moreover, controlling the transcriptional regulation of genes through gene editing could be an alternate strategy for trait improvement. Advancements in CRISPR/Cas9 technology would help to know gene expression regulation in a better way and provide an additional tool. Keeping in mind that excessive Fe concentration causes oxidative stress in plants, future attempts for the modulation of Fe homeostasis may consider both negative and positive regulators, including new players, such as IRO3, HRZ, and IMA, in wheat (Kobayashi et al., 2013; Grillet et al., 2018). Moreover, to control excessive Fe loading into the plant, the gene expressions can be fine-tuned with the use of CRISPRi and/or CRISPRa. For this, several vectors and toolkits with various monocot-specific strong promoters and gene regulatory elements (activators/repressors) are available in the Addgene repository (Kamens, 2015). The expansion of wheat genomic resources (Borrill et al., 2016; Appels et al., 2018; Ramírez-González et al., 2018) along with the modern gene-editing tools *via* CRISPR/Cas9 could offer a reliable potential to achieve Fe-rich wheat grains.

## Author Contributions

AP and PB conceived and designed the review article. AK, PS, and MT contributed and collated information for the review article. GK, PS, SS, AP, and VM designed the figure and wrote the initial draft. GK, MT, and PB contributed for Tables. AP, MT, and PB edited the manuscript. All authors agreed with the final version.

## Conflict of Interest

The authors declare that the research was conducted in the absence of any commercial or financial relationships that could be construed as a potential conflict of interest.

## Publisher’s Note

All claims expressed in this article are solely those of the authors and do not necessarily represent those of their affiliated organizations, or those of the publisher, the editors and the reviewers. Any product that may be evaluated in this article, or claim that may be made by its manufacturer, is not guaranteed or endorsed by the publisher.
